# Emergency Appendectomy during the Early Phase after ^177^Lu-DOTATATE Therapy: A Case Report with Perioperative Radiation Safety after PRRT

**DOI:** 10.70352/scrj.cr.26-0286

**Published:** 2026-06-18

**Authors:** Kei Nakagawa, Tomoki Fukata, Hiroto Sakurai, Kenichiro Yambe, Noriko Kondo, Kazuhiro Takami, Kuniharu Yamamoto, Yu Katayose

**Affiliations:** Division of Hepato-Biliary and Pancreatic Surgery, Tohoku Medical and Pharmaceutical University, Sendai, Miyagi, Japan

**Keywords:** Lutetium-177–DOTATATE, peptide receptor radionuclide therapy, pancreatic neuroendocrine tumor, acute appendicitis

## Abstract

**INTRODUCTION:**

Peptide receptor radionuclide therapy (PRRT) with ^177^Lu-DOTATATE has become an established treatment for somatostatin receptor-positive neuroendocrine tumors. However, the safety of performing emergency surgery during the early post-treatment period remains unclear, given concerns regarding radiation exposure and potential contamination. We report a case of acute appendicitis requiring emergency surgery shortly after PRRT, with a focus on perioperative radiation safety.

**CASE PRESENTATION:**

A 75-year-old man with a pancreatic neuroendocrine tumor and multiple liver metastases underwent PRRT at another institution. Six days after treatment, he developed acute appendicitis and was referred to our hospital. Laboratory findings showed leukocytosis and elevated inflammatory markers. Emergency laparoscopic appendectomy was performed, and intraoperative radiation dose rates were measured using a survey meter. At a distance of 1 m from the patient, radiation levels were low (1.1 μSv/h), while measurements at 20 cm from the operative field ranged from 8.4 to 12.2 μSv/h, remaining within acceptable safety limits. Radiation levels detected in urine were also low (0.2 μSv/h at 5 cm). The postoperative course was uneventful, and the patient was discharged on POD 4 without complications.

**CONCLUSIONS:**

This case demonstrates that emergency surgery can be performed safely during the early phase after PRRT when appropriate radiation safety precautions are implemented. Radiation exposure to surgical staff remained minimal at typical working distances, and even near-field exposure was within acceptable limits. Urgent surgical intervention should not be unnecessarily delayed following PRRT when clinically indicated, provided that appropriate monitoring and precautions are in place.

## Abbreviations

pNET
: pancreatic neuroendocrine tumor
PRRT
: peptide receptor radionuclide therapy
SSTR
: somatostatin receptor

## INTRODUCTION

pNETs frequently present with liver metastases.^1,2)^ Treatment options include resection of the primary tumor and hepatic lesions, as well as chemotherapy and PRRT. In recent years, studies have demonstrated the efficacy of PRRT,^3)^ and the number of facilities offering this treatment has increased in Japan. However, availability remains limited, and waiting periods are often prolonged; consequently, many patients undergo PRRT at facilities located far from their homes.

In this report, we describe an appendectomy performed in a patient who developed acute appendicitis on the sixth day after undergoing PRRT at a facility that had not yet introduced this therapy. We confirmed the therapeutic half-life and used a survey meter to assess patient safety and evaluate the potential for radiation contamination of excreta and other materials. This clinical experience provides practical insights into the management of emergency conditions following PRRT, a treatment modality that is becoming increasingly common.

## CASE PRESENTATION

A 75-year-old man, 167 cm in height and weighing 64.5 kg, presented for evaluation. Family history included colorectal cancer in his mother. Lifestyle history included consumption of approximately 1000 mL of beer once weekly and smoking 20 cigarettes per day for more than 40 years.

The patient had been receiving treatment for hypertension at another hospital. In Year X–6, abnormal liver function test results prompted CT, which revealed a tumor in the pancreatic tail and multiple hepatic lesions. These findings led to referral to the Department of Gastroenterology at our hospital for further evaluation.

The pancreatic tail tumor demonstrated internal calcifications and partial enhancement on CT (**Fig. 1A**), and the hepatic lesions also showed internal enhancement (**Fig. 1B** and **1C**). On MRI, the pancreatic tumor appeared hypointense on T2-weighted images. Based on these imaging findings, liver metastases from a pNET were suspected.

**Fig. 1:**
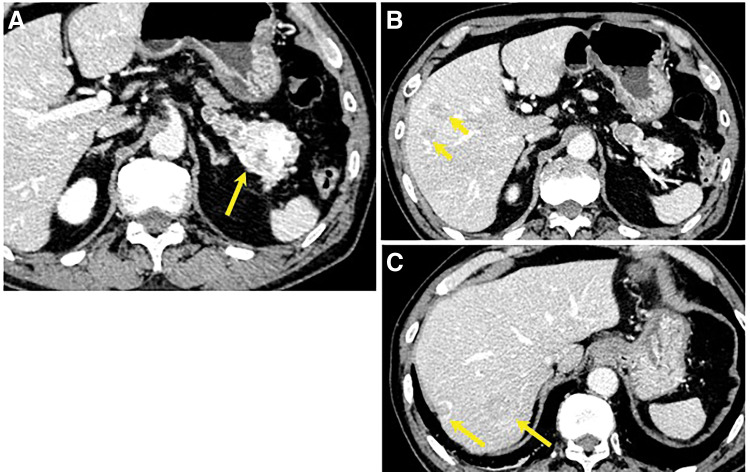
CT at the initial visit in Year X–6. (**A**) Pancreatic tail tumor with low enhancement and internal calcifications. (**B**, **C**) Multiple hepatic lesions with internal enhancement in both lobes.

MRI more clearly delineated the hepatic lesions, identifying 8 tumors (**Fig. 2A**). An Octreoscan (Fujifilm RI Pharma Co., Ltd., Tokyo, Japan) using indium-111 pentetreotide was subsequently performed (**Fig. 2B**). Strong mass-like uptake was observed in the pancreatic tail, consistent with a neuroendocrine tumor. The 5-h images demonstrated multiple nodular areas of uptake in both hepatic lobes, which became more pronounced on the 24-h images, consistent with metastatic liver disease.

**Fig. 2:**
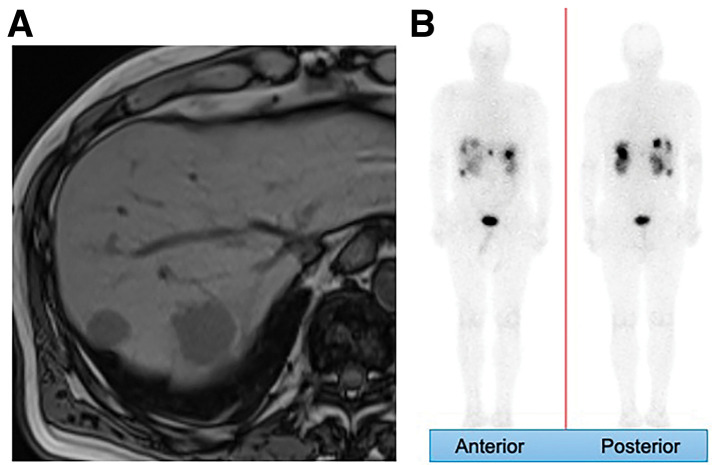
MRI and Octreoscan findings of pNET and liver metastases. (**A**) MRI clearly delineated the hepatic lesions, confirming 8 tumors. (**B**) Five-hour Octreoscan (SSTR imaging) showing uptake in both the primary tumor and liver metastases. pNET, pancreatic neuroendocrine tumor; SSTR, somatostatin receptor

Endoscopic US-guided tissue acquisition was performed for the pancreatic tumor. Histopathological examination revealed a proliferating tumor with stromal components (**Fig. 3A**). Immunohistochemistry showed that the tumor cells were positive for cytokeratin (AE1/AE3), chromogranin A, synaptophysin, and CD56, confirming the diagnosis of a pNET.

**Fig. 3:**
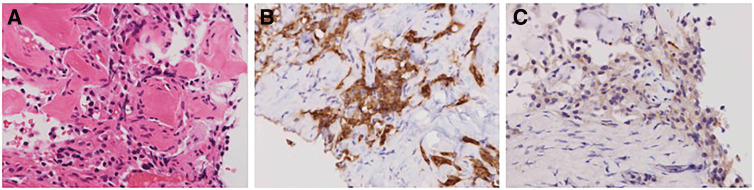
Histopathological features of the pancreatic tumor on endoscopic US-guided tissue acquisition. (**A**) Hematoxylin and eosin staining showing a proliferating tumor with stromal components. (**B**) SSTR subtype 2 score of 3. (**C**) SSTR subtype 5 score of 1. SSTR, somatostatin receptor

The mitotic count was approximately 0–1 per 10 high-power fields, and the Ki-67 index was around 2%. Although definitive grading remained limited by the small tissue sample, the tumor was classified as grade 1 or grade 2. The tumor cells were positive for pancreatic polypeptide and negative for serotonin, glucagon, insulin, somatostatin, and gastrin. In addition, SSTR expression corresponded to an SSTR2 score of 3 (**Fig. 3B**) and an SSTR5 score of 1 (**Fig. 3C**).

Treatment with the somatostatin analog lanreotide was initiated. Although the disease remained controlled for a period, a CT scan in September of Year X–1 showed increased contrast enhancement within the hepatic lesions (**Fig. 4**). The patient subsequently inquired at another hospital regarding the possibility of PRRT, and treatment was scheduled approximately 3 months later. A CT scan was performed on January 20, Year X, prior to PRRT (**Fig. 5**). The patient was admitted to the referring hospital on January 22 and underwent PRRT.

**Fig. 4:**
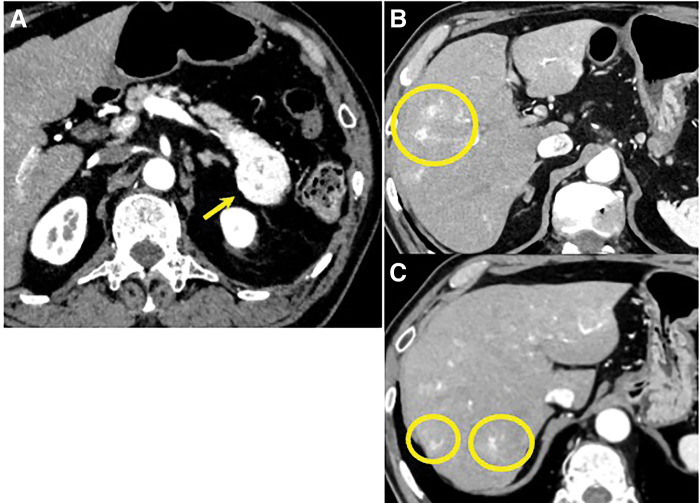
CT 5 years after somatostatin analog therapy. (**A**) No significant change in the pancreatic tumor during follow-up. (**B**, **C**) Hepatic lesions showing regrowth after initial reduction, with increased contrast enhancement.

**Fig. 5:**
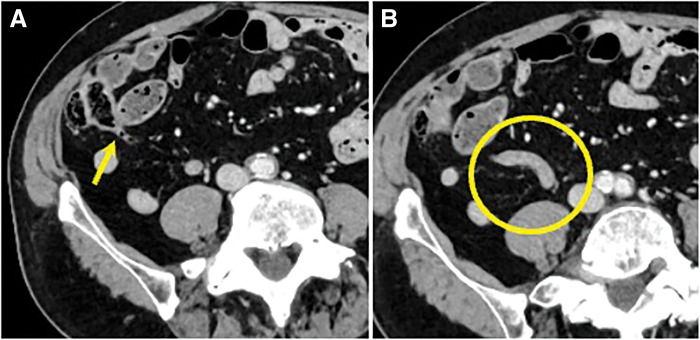
CT prior to PRRT for evaluation of pancreatic and hepatic lesions. (**A**) Base of the appendix showing intraluminal air without focal abnormalities. (**B**) Mild enlargement of the appendiceal body. PRRT, peptide receptor radionuclide therapy

After discharge, the patient developed abdominal pain on January 28 and presented to our emergency department on January 29. CT revealed enlargement of the appendix and the presence of intraluminal calculi compared with the scan performed on January 20, leading to a diagnosis of acute appendicitis (**Fig. 6**). Laboratory results were as follows: white blood cell count, 21800/μL; hemoglobin, 13.9 g/dL; platelet count, 334000/μL; total bilirubin, 1.24 mg/dL; aspartate aminotransferase, 20 U/L; alanine aminotransferase, 19 U/L; lactate dehydrogenase, 221 U/L; alkaline phosphatase, 73 U/L; γ-glutamyl transferase, 64 U/L; and C-reactive protein, 3.95 mg/dL.

**Fig. 6:**
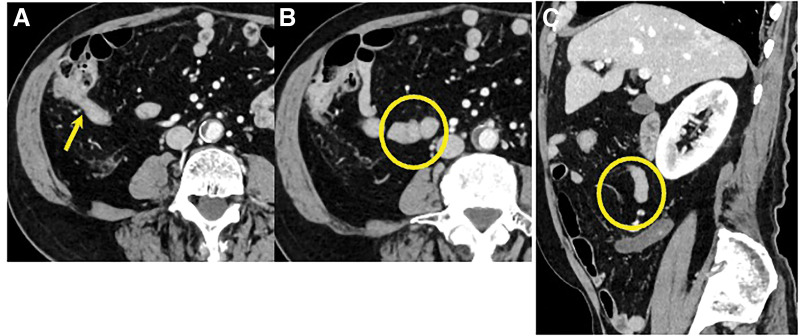
CT findings at the onset of abdominal pain. (**A**) Appendiceal base showing marked enlargement. (**B**) Axial view of the appendiceal body showing enlargement. (**C**) Sagittal view of the appendiceal body showing enlargement, consistent with appendicitis. Appendicolith was also observed.

The marked leukocytosis, elevated C-reactive protein level, progressive abdominal pain, and presence of appendicular enlargement and appendicolith suggested a risk of disease progression. We concluded that emergency surgery was appropriate in this case, given the risk of systemic inflammation and disease progression.^4)^

We confirmed that the therapeutic half-life following PRRT had elapsed and therefore considered the risk of radiation exposure to be low. In addition, concern for complications such as perforation or peritonitis, particularly in the setting of potential post-treatment pancytopenia, supported prompt intervention. In light of these considerations, we decided to perform an appendectomy. Additionally, although extremely rare, we attempted to resect the appendix down to its base in anticipation of the possibility that pNET metastasis to the appendix might have caused the inflammation following PRRT treatment.

The procedure was performed laparoscopically (**Fig. 7**). The appendix was enlarged and adherent to surrounding structures, with no evidence of infiltration. Partial cecal resection was performed to ensure adequate margins. The operative time was 134 min, and blood loss was minimal.

**Fig. 7:**
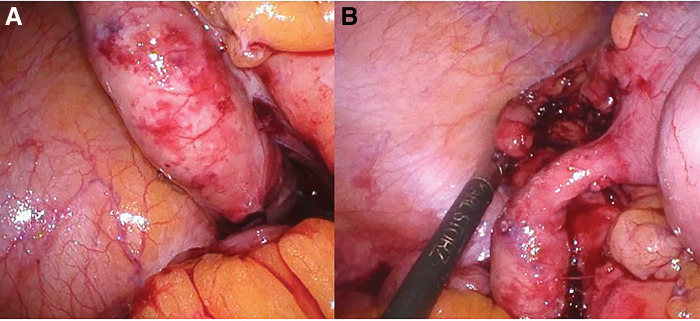
Intraoperative findings of the appendix. (**A**) Appendiceal body adherent to surrounding tissues without evidence of tumor infiltration. (**B**) Appendiceal base resected with a portion of the cecal wall.

Following induction of general anesthesia, radiation doses were measured using an ionization chamber survey meter (ICS-321; ALOKA Co., Ltd., Tokyo, Japan). We consulted with the radiation oncologist and confirmed that, based on the half-life, the level of contamination on the body surface and in body fluids was low and would not interfere with the surgery. Furthermore, to ensure safety, we measured radiation levels preoperatively using a calibrated dosimeter. Since the measured levels were low, no shielding measures were necessary. Based on previous treatment reports, it was predicted that there was virtually no possibility of high radiation levels being detected. However, in the unlikely event that unthinkably high levels were detected—specifically, approximately 20–30 μSv/h at a distance of 1 m—we had planned to perform a brief surgical procedure while wearing radiation protective clothing. If the radiation level exceeded 30 μSv/h, we planned to cancel the surgery and consider conservative treatment, postponement of surgery, or transfer of the patient to a facility equipped with radiation shielding. In Japan, the practical discharge criteria following I-131 radionuclide therapy are based on a dose rate of 30 μSv/h or less, measured at a distance of 1 m from the patient, or residual radioactivity of 500 MBq or less.

Antibiotics were administered for 3 days postoperatively. Although the C-reactive protein level increased to 16.6 mg/dL on the first POD, both the white blood cell count and C-reactive protein level subsequently returned to normal. The patient recovered uneventfully and was discharged on POD 4. Pathological examination of the resected appendix revealed no evidence of neuroendocrine tumor or malignancy. The second PRRT session was administered on March 19, and the patient continues to do well.

In the present case, radiation dose rates demonstrated a clear distance-dependent decline, consistent with the inverse square law, as illustrated in **Fig. 8**. Although measurable radiation levels were observed at 20 cm from the patient (8.4–12.2 μSv/h), these values represent a worst-case scenario simulating close proximity to the operative field. Importantly, the dose rate at a distance of 1 m was as low as 1.1 μSv/h, approaching the lower range of environmental background exposure. Furthermore, radioactivity detected in urine, even at a close distance of 5 cm, remained minimal (0.2 μSv/h), suggesting a limited risk of contamination from body fluids under standard precautions.

**Fig. 8:**
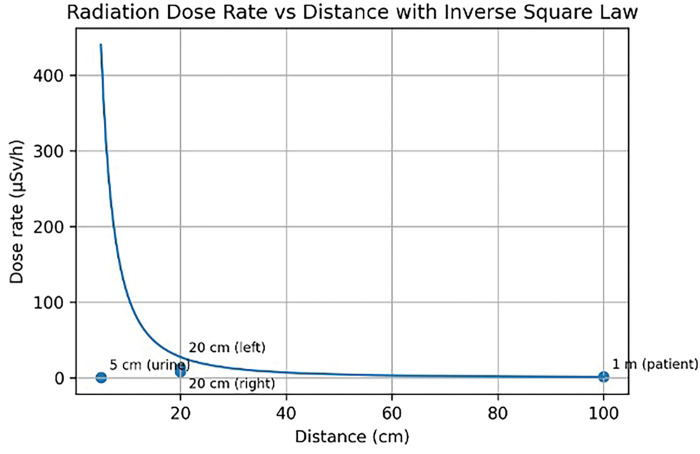
Radiation dose distribution around the patient following PRRT based on distance measurements. Radiation dose rates measured at various distances from the patient and the urine sample, with a theoretical curve based on the inverse square law. Measurements at 20 cm represent a worst-case exposure scenario near the operative field, whereas dose rates at 1 m were minimal. The distribution demonstrates rapid attenuation of radiation with increasing distance. PRRT, peptide receptor radionuclide therapy

The concordance between the measured values and the theoretical inverse square curve supports the validity of the measurements and indicates rapid attenuation of radiation with increasing distance. Taken together, these findings suggest that radiation exposure to surgical staff during emergency procedures performed approximately one half-life after ^177^Lu-DOTATATE administration is likely to be clinically negligible, particularly at typical working distances. Therefore, urgent surgical intervention should not be delayed solely based on recent PRRT, provided that appropriate radiation safety measures are implemented.

## DISCUSSION

pNETs are rare, with an annual incidence of approximately 0.2–1.0 cases per 100000 individuals, accounting for 1%–2% of all pancreatic tumors.^5–7)^ Their incidence has increased markedly in recent decades, largely reflecting advances in imaging and the detection of incidental lesions. Most pNETs are nonfunctioning and are frequently diagnosed at an advanced stage, with a substantial proportion presenting with metastatic disease, particularly involving the liver.

Nonfunctioning pNETs often remain asymptomatic, which contributes to delayed diagnosis and a high rate of liver metastasis. Although the likelihood of hepatic metastasis increases with higher Ki-67 indices, metastases occur in 30%–40% of cases even in grade 1 tumors. In contrast, prognosis varies significantly by grade, with reported 5-year survival rates of 91% for grade 1%, 70.8% for grade 2%, and 20% for grade 3 tumors,^8)^ supporting the need for long-term management strategies in these patients.

If liver metastases are localized and R0/R1 resection or adequate debulking is feasible, hepatic resection remains the standard of care.^1,2)^ For SSTR-positive grade 1 or grade 2 pNETs with minimal symptoms and indolent progression, somatostatin analogs are often selected as first-line therapy. In contrast, cases with high tumor burden, clear disease progression, or progression after somatostatin analog therapy require escalation to PRRT, targeted therapy, or chemotherapy, administered sequentially or earlier depending on clinical factors.

^177^Lu-DOTATATE is a radiolabeled somatostatin analog used in PRRT for patients with SSTR-positive neuroendocrine tumors. The radionuclide lutetium-177 has a physical half-life of approximately 6.7 days and is primarily excreted via the kidneys. Hematologic toxicity, including bone marrow suppression, represents a recognized adverse effect of this therapy.^9)^

The phase III NETTER-2 trial demonstrated that first-line PRRT significantly prolonged progression-free survival compared with high-dose octreotide in patients with newly diagnosed, SSTR-positive grade 2 and grade 3 gastroenteropancreatic neuroendocrine tumors. These findings support a shift toward earlier use of PRRT, particularly in patients with higher-grade disease defined by a Ki-67 index of 10% or greater.^3)^ Accordingly, PRRT represents an appropriate treatment option for a substantial proportion of patients with pNET.

Hematologic toxicity, including bone marrow suppression, also represents a recognized adverse effect of this therapy. These characteristics raise important concerns regarding the safety of performing invasive procedures, particularly emergency surgery, during the early post-treatment period.^10,11)^ Hematologic toxicity typically occurs not immediately after treatment, but 2–6 weeks after PRRT. If peritoneal inflammation develops at this time, there is a risk of rapid progression to sepsis or disseminated intravascular coagulation. However, given individual variability and the possibility of delayed cytopenia, we took the risk of bone marrow suppression into account when making our decision.

From a radiation protection perspective, PRRT requires dedicated facilities that comply with radiation safety regulations, including shielded patient rooms and appropriate handling of radiopharmaceuticals. Because ^177^Lu-DOTATATE is primarily excreted in the urine, radioactive contamination of body fluids requires careful management, and waste disposal must follow institutional and national guidelines.^12)^ Consequently, patients who have recently undergone PRRT are temporarily considered radiation sources, and invasive procedures performed shortly after administration may theoretically expose surgical staff to ionizing radiation.

Despite these concerns, data on the safety of emergency surgery during the early period after PRRT remain limited. Most of the available literature focuses on treatment efficacy and systemic toxicity, while perioperative management in this context remains largely unaddressed. To our knowledge, reports describing emergency abdominal surgery, particularly appendectomy, shortly after PRRT are extremely scarce. Accordingly, clinical decision-making in such situations relies on theoretical considerations, including radionuclide decay, excretion kinetics, and general radiation safety principles.

In the present case, the measured radiation dose rates were low and considered clinically acceptable. The maximum dose rate observed at 20 cm from the patient was 12.2 μSv/h, representing a worst-case exposure scenario corresponding to close proximity to the operative field. At a distance of 1 m, the dose rate was as low as 1.1 μSv/h, which is close to background radiation levels. According to international radiation protection guidelines, the annual occupational exposure limit for medical personnel is 20 mSv per year, averaged over defined periods. Even assuming continuous exposure at the highest measured level (12.2 μSv/h), the cumulative dose during a typical surgical procedure would remain extremely low. For example, during a 2-h operation, the estimated exposure would be approximately 24 μSv, which is less than 0.2% of the annual occupational dose limit. In the present case, emergency appendectomy was performed 7 days after administration of ^177^Lu-DOTATATE, corresponding to approximately one physical half-life of the radionuclide. In contrast, delaying surgery carried a risk of disease progression, including perforation or peritonitis.

Intraoperative radiation measurements demonstrated that exposure levels remained within acceptable safety limits for medical personnel. These findings indicate that, with appropriate precautions, emergency surgery can be performed safely during the early post-PRRT period. Radioactivity decreases exponentially over time, and a substantial proportion of the radiopharmaceutical is eliminated through urinary excretion within the first several days. Consequently, the actual radiation risk to operating staff may be lower than initially anticipated.

On the other hand, in cases where radiation levels are actually high—such as on the day after treatment—it is necessary to consider treatment strategies for emergency conditions.

Specifically, if the radiation dose rate at 1 m exceeds generally accepted thresholds (e.g., 18–30 μSv/h), initial conservative treatment with antibiotics is first attempted. If the patient’s condition permits, based on the outcome of that treatment, surgery may be postponed until radiation levels decrease. However, if conditions such as gastrointestinal perforation, peritonitis, or sepsis arise, making emergency surgery unavoidable, surgery is performed after implementing enhanced radiation protection measures (e.g., shortening the duration of surgery, maintaining distance, and using shielding materials). In such cases, it is necessary to measure the radiation dose of excised organs, blood, and bodily fluids such as peritoneal lavage fluid and urine, and to isolate them.

Our findings have several clinical implications. First, urgent surgical intervention should not be delayed solely based on recent PRRT when clinically indicated. Second, intraoperative radiation monitoring can provide objective reassurance regarding staff safety. Third, adherence to fundamental radiation protection principles—minimizing exposure time, maximizing distance, and using appropriate shielding—remains essential. Finally, multidisciplinary collaboration among surgeons, nuclear medicine specialists, and radiation safety personnel is critical in managing such complex cases.

This report has some limitations. As a single case, the findings have limited generalizability, and radiation exposure may vary according to tumor burden, renal function, and the interval after PRRT. Further accumulation of similar cases and systematic evaluation of perioperative radiation exposure are needed to establish evidence-based guidelines.

## CONCLUSIONS

Even after a short period of isolation following PRRT, caution regarding radioactive excretion remains necessary. At the same time, potential complications such as pancytopenia warrant consideration, and conservative management may exacerbate conditions such as acute appendicitis or cholecystitis. This case demonstrates that appropriate surgical intervention for emergency conditions following PRRT can be performed without significant radiation exposure to medical staff. However, because renal excretion varies among individuals, direct measurement using a survey meter, as performed in this case, provides a practical and effective approach to confirm safety.

## DECLARATIONS

### Availability of data and materials

All data generated or analyzed during this study are included in this published article.
